# CDK7 inhibition promotes glioblastoma cell death through convergent transcriptional and metabolic stress pathways

**DOI:** 10.1002/ctm2.70448

**Published:** 2025-08-20

**Authors:** Thomas Freitag, Jana Vanessa Scheffler, Philipp Kaps, Anne Hieltscher, Björn Schneider, Daniel Dubinski, Florian Gessler, Thomas M. Freiman, Felix Wittig, Sascha Troschke‐Meurer, Wendy Bergmann‐Ewert, Susanne Staehlke, Lars Boeckmann, Philipp‐Kjell Ficht, Christian Polley, Christian Junghanss, Burkhard Hinz, Claudia Maletzki

**Affiliations:** ^1^ Department of Medicine, Clinic III – Hematology Oncology, Palliative Medicine, Rostock University Medical Center, University of Rostock Rostock Germany; ^2^ Department of Neurosurgery Rostock University Medical Center, University of Rostock Rostock Germany; ^3^ Institute of Pathology, Rostock University Medical Center, University of Rostock Rostock Germany; ^4^ Institute of Pharmacology and Toxicology, Rostock University Medical Center, University of Rostock Rostock Germany; ^5^ Department of Pediatric Oncology and Hematology University Medicine Greifswald Greifswald Germany; ^6^ Core Facility for Cell Sorting & Cell Analysis, Rostock University Medical Center, University of Rostock Rostock Germany; ^7^ Institute of Cell Biology, University Medical Center Rostock Rostock Germany; ^8^ Clinic and Policlinic for Dermatology and Venereology, University Medical Center Rostock Rostock Germany; ^9^ Department of Microfluidics, Faculty of Mechanical Engineering and Marine Technology, University of Rostock Rostock Germany

1

Dear Editor

Cyclin dependent kinase 7 (CDK7) is frequently overexpressed in various malignancies, including glioblastoma (GBM), where elevated expression correlates with poor prognosis.^1^, ^2^, ^3^ Here, we report that the covalent CDK7 inhibitor, THZ1^4^, ^5^ suppresses GBM proliferation by impairing mitochondrial function and activating the unfolded protein response (UPR), resulting in autophagy and non‐apoptotic cell death, characterised by features consistent with methuosis.^6^ In patient‐derived GBM organoids (PDOs^7^) with different molecular subtypes, THZ1 significantly reduced viability. Mechanistically, THZ1 treatment disrupted key cellular pathways, including metabolism, immune signalling, RNA processing, and cell adhesion. We also identified intrinsic resistance associated with calcium signalling and mitochondrial regulation in a rare GBM subtype.

THZ1 has limited clinical utility due to poor metabolic stability, susceptibility to ABC transporter‐mediated efflux, and restricted blood‐brain barrier (BBB) penetration; however, it remains a valuable tool for elucidating CDK7‐dependent transcriptional vulnerabilities in GBM.^5^ Importantly, CDK7 inhibition demonstrated potent anti‐tumour effects in preclinical GBM models, independently of MGMT promoter methylation status, by disrupting transcription, inducing apoptosis, and suppressing stemness.^1^, ^2^, ^3^, ^4^ Mevociclib, a more stable CDK7 inhibitor, recapitulated THZ1‐associated effects, including lysosomal activation and reduced invasion, further underscoring CDK7 as a therapeutic target in GBM.

Firstly, dose‐response analyses on four patient‐derived GBM cell lines (GBM03, GBM06, GBM14, and GBM15) with distinct molecular features, including methylated and unmethylated MGMT promoters (Table 1), revealed nanomolar‐range sensitivity to THZ1 (Figure 1A), accompanied by DNA damage response activation and apoptosis (Figure 1B). Despite cell loss, residual cells retained nascent protein synthesis, indicating stress‐induced translational adaptation (Figure S1A and B). Mitochondrial dysfunction emerged as a key mechanism, evidenced by membrane potential loss, increased permeability, elevated ROS, and significantly reduced basal respiration, ATP production, spare respiratory capacity, and proton leak in Seahorse assays (Figure 1C–E). These metabolic defects persisted 10 days post‐treatment, indicating sustained failure to restore energy metabolism (Figure 1F). This was accompanied by altered mitochondrial morphology, increased lysosomal activity, and endoplasmic reticulum (ER) stress with concurrent UPR activation^8^ (Figures 1G and H and S1E and F). Interestingly, Mevociclib elicited less ER stress but similarly increased lysosomal activity (Figure S2A and B), supporting autophagy induction through distinct yet converging mechanisms. THZ1 treatment led to a marked upregulation of the UPR sensors IRE1 and PERK in both GBM06 and GBM15 cells, while ATF6 induction was observed only in GBM06 (Figure S1E and F). These findings indicate that THZ1‐induced ER stress primarily involves activation of the IRE1–PERK axis and may contribute to the resulting cellular dysfunction and death.

**TABLE 1:** Clinical patients data, including basic MGMT methylation status in different GBM samples used for PDO establishment.

Patient ID	Gender/age	Localisation	*MGMT* status	Histology	Mutational profile
GBM06	m/71	Temporal lobe	Methylated	GFAP^+^/MAP2^+^, ATRX preserved	*TP53* G244A (VAF: 99.9%) P72R (VAF: 100.0%) *PIK3CA* I391M (VAF: 30.6%)
GBM15	m/40	Left parietal	Unmethylated	GFAP^+^/MAP2^+/−^, ATRX preserved	*TP53* P72R (VAF: 99.9%)
GBM23	m/73	Right temporofrontal lobe	Methylated	GFAP^+^	N/A
GBM26	w/72	Right occipitotemporal lobe	Methylated	GFAP^+^/MAP2^+^, ATRX preserved	*ATM* R2443^*^ (VAF: 51.6%)
GBM31	w/61	Right frontal lobe	Unmethylated	GFAP^+^/MAP2^+^, ATRX preserved	N/A
GBM36^#^	m/46	N/A	Methylated		N/A
GBM37	w/74	Multifocal	Methylated		N/A
GBM39	m/58	Left temporal lobe	Unmethylated	ATRX preserved, H3.3K27M loss	*TP53* R342^*^ (VAF: 81.2%)
GBM40	m/68	Left parietal	Unmethylated		N/A
GBM41	m/58	Pons	Unmethylated		*PIK3CA* E545K (VAF: 50%) *PTEN* M134V (VAF: 56.7%) *TP53* C135R (VAF: 64.0%)

m, male; f, female.

^#^ Oligodendroglioma.

* Stop codon.

**FIGURE 1:**
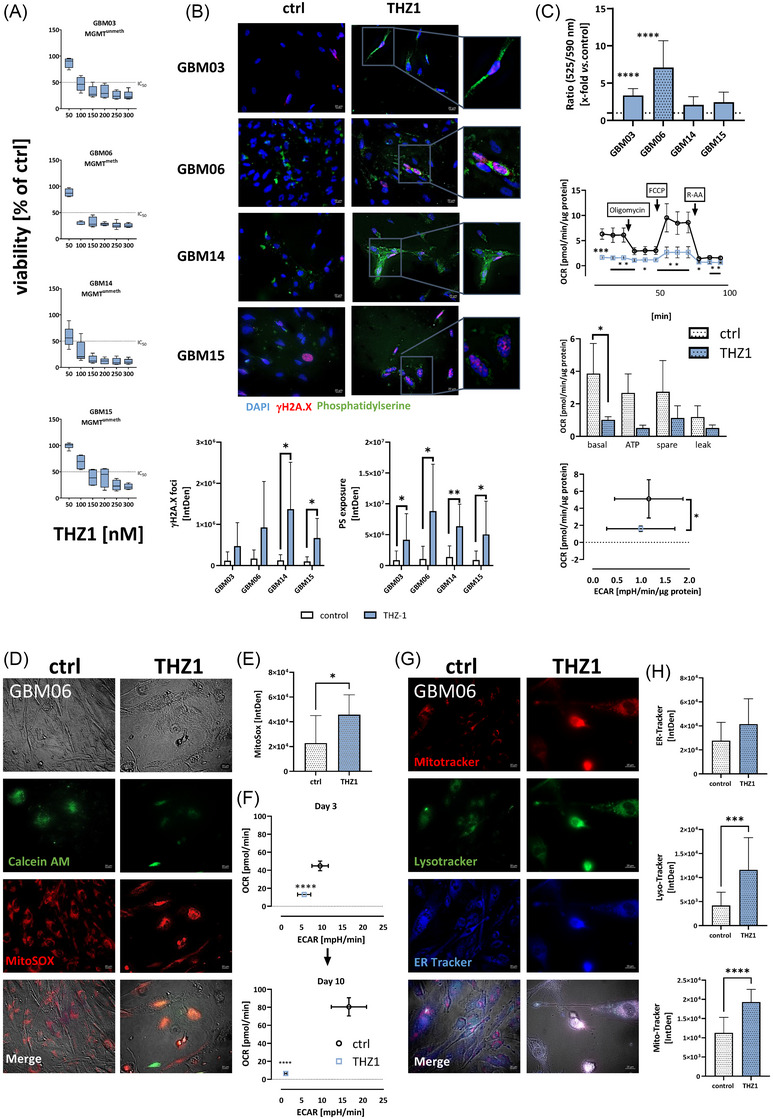
Effect of CDK7 blockade by THZ1 on GBM cells. (A) Concentration‐response relationships to THZ1 after two 72 h treatment cycles to determine the IC50 concentration. (B) Effect of THZ1 on DNA damage (γH2A.X) and apoptosis (phosphatidylserine) after two treatment cycles with 72 h each (GBM03, GBM06: 75 nM THZ1; GBM14, GBM15: 100 nM THZ1). Representative images of double‐strand breaks are shown on the upper part, obtained using Alexa Fluor® 594‐conjugated anti‐H2A.X Phospho (Ser139) antibody. Apotracker green was used to detect apoptosis. Scale bar indicates 20 µm. Quantitative analysis of the integrated density for the DNA damage marker γH2A.X and the apoptosis marker Phosphatidylserine. *N* ≥ 3, mean + SD, *t*‐test; *p < .05; **p < .01 vs. control. (C–G) Impact of CDK7 blockade on mitochondrial fitness, lysosome formation, and extracellular flux analysis. (C) JC‐10 Mitochondrial Membrane Potential Assay and Seahorse analysis. The oxygen consumption rate (OCR) and extracellular acidification rate (ECAR) of GBM06 cells were measured using a Seahorse XFe24 Analyzer. *N* ≥ 3, mean + SD, *t*‐test; *p < .05; **p < .01; ***p < .001 ****p < .0001 vs. control. Mitochondrial stress test was applied with injection of 1.5 µM oligomycin, 1.5 µM FCCP and 0.5 µM antimycin A and rotenone, each. *p < .05; **p < .01; ***p < .001; ****p < .0001 vs. control. To determine the basal respiration (basal), ATP dependent respiration (ATP), Spare respiratory capacity (spare) and proton leak (leak) were determined. *N* ≥ 3, mean + SD, *t*‐test; *p < .05 vs. control. Reduction of basal OCR and spare respiratory capacity was shown, indicating massively reduced cellular fitness. *N* ≥ 3, mean + SD, *t*‐test; ****p < .0001 vs. control. (D, E) MitoSOX™ red was utilised to indicate ROS production in GBM cells, while Calcein AM [green] was used for counterstaining to visualise spatial distribution. (D) Fluorescence images are shown as single‐channel and merged views. Scale bar indicates 20 µm. (E) Quantification of the integrated density; *n* ≥ 3, mean + SD, *t*‐test; *p < .05 vs. control. Dotted pattern – MGMT^meth^. (F) OCR of GBM06 cells after 3 days and 10 days after single treatment with THZ1 (75 nM) (G, H) ER stress (ER tracker), acidic compartments (LysoTracker), and mitochondrial function (Mitotracker) were examined in 2D‐cultured GBM cells with or without treatment (THZ1 75 nM) by immunofluorescence staining as described in materials and methods (ER‐ [blue], Lyso‐ [green], and Mitotracker [red]). (G) Single channel and merged fluorescence are presented (representative images are shown, scale bar: 20 µm). Scale bar indicates 20 µm. (H) Quantification of the integrated density; *n* ≥ 3, mean + SD, *t*‐test; ***p < .001; ****p < .0001 vs. control. Dotted pattern – MGMT^meth^.

We next examined THZ1‐induced stress responses in GBM06 and GBM15 cells. THZ1 significantly increased LAMP‐1^+^/Rab7a^+^ cells, indicating late endosomes associated with methuosis (Figure 2A). This was corroborated by immunofluorescence, which confirmed LAMP‐1/Rab7a co‐localisation, distinguishing this process from typical endosomal or lysosomal swelling (Figure 2B). Residual cells also exhibited elevated LC3B^+^ levels, revealing autophagy (Figure 2A). To assess autophagic flux, we analysed LC3B⁺ vesicles with and without Bafilomycin A1 (BafA1), an inhibitor of lysosomal acidification and autophagosome–lysosome fusion. The accumulation of LC3B⁺ structures indicated impaired degradation rather than increased autophagy. Similarly, the rise in LAMP‐1⁺/Rab7a⁺ vesicles with BafA1 co‐treatment indicated defective vesicle clearance rather than enhanced methuotic activity.

**FIGURE 2:**
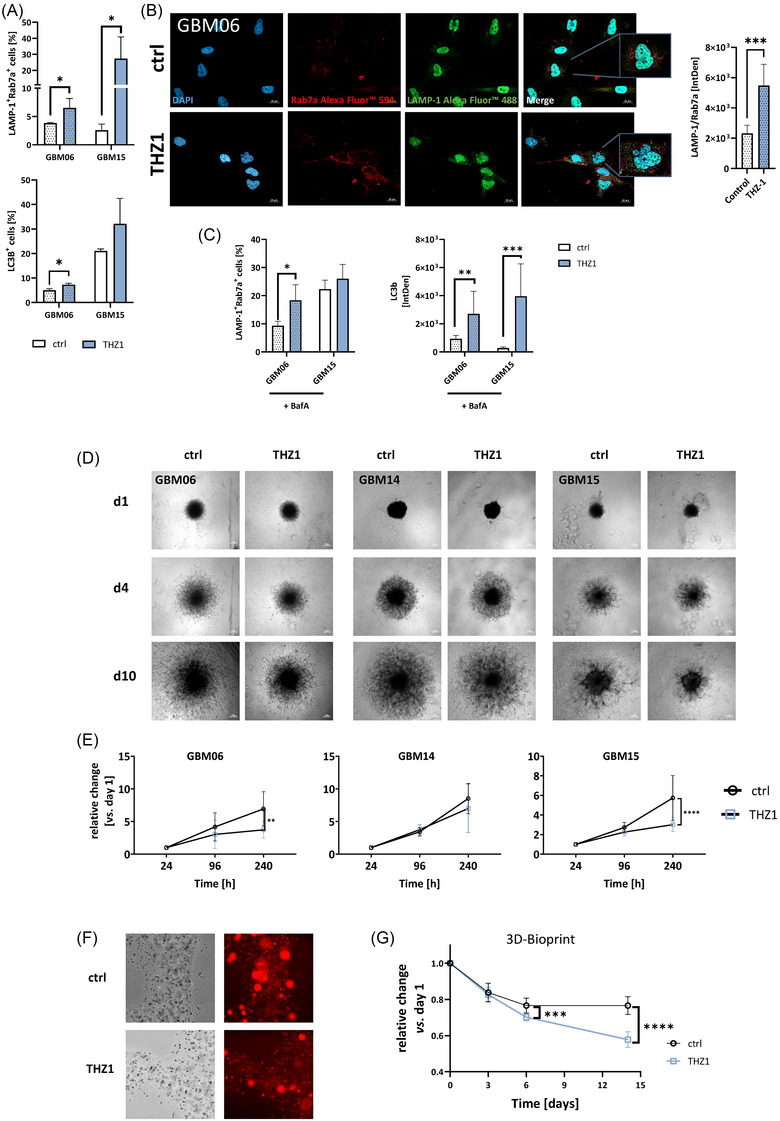
Influence of CDK7 blockade on autophagy and methuosis in 2D culture and invasive and viability in 3D culture. (A) Quantitative analysis of the investigated markers using spectral flow cytometry. Analysis was performed with GBM06 and GBM15 cells after 2 × 72 h with or without treatment (GBM06: 75 nM THZ1, GBM15: 100 nM THZ1). Given is the percentage of positive cells after treatment; *n* ≥ 3, mean + SD, *t*‐test; *p < .05 vs. control. (B, C) Immunofluorescence analysis of methuosis. (B) Cells stained with Alexa Fluor® 594 anti‐Rab7a (red), FITC anti‐LAMP‐1 (green), and DAPI (blue) are shown as single‐channel and merged fluorescence images. Scale bar indicates 20 µm. Quantitative analysis of integrated density; *n* ≥ 3, mean + SD, *t*‐test; ***p < .001 vs. control. (C) To distinguish methuosis from apoptosis, bafilomycin A1 was added with or without treatment (GBM06: 75 nM, GBM15: 100 nM) to selectively identify methuosis‐positive cells; Quantitative analysis was done using spectral flow cytometry or immunofluorescence on GBM06 and GBM15 cells treated for 2 × 72 h with or without THZ1 (GBM06: 75 nM, GBM15: 100 nM). N ≥ 3, mean ± SD, *t*‐test; *p < .05; **p < .01; ***p < .001 vs. control. Dotted pattern – MGMT^meth^. (D–G) Invasion and viability in 3D‐spheroid and bioprinting models. (D) Representative images of the invasion under treatment with THZ1 (GBM06: 75 nM THZ1, GBM14, GBM15: 100 nM THZ1) or with vehicle control after the spheroids were embedded in matrigel. Images were taken on day 1, 4 and 10. The spheroids were treated on day 0 and day 3 (2 × 72 h in total). (E) Relative spheroid size compared to day 1, which was set to 1. *N* ≥ 3, mean ± SD, *t*‐test; **p < .01; ****p < .0001 vs. control. Scale bar indicates 50 µm. (F, G) A 3D‐bioprinting approach was used to analyse the impact of THZ1 on GBM cell growth in biomimetic scaffolds. Therefore, cell‐laden scaffolds were treated with THZ1 or vehicle control for 2 × 72 h. Viability was assessed longitudinally by ImageXpress high content analysis. Representative images of the scaffolds at day 14 (Original magnification 200×). (F) Longitudinal assessment of GBM cell viability in 3D‐bioprinted scaffolds treated with THZ1 (100 nM) over 10 days. Cells were stably transduced to express a near‐infrared fluorescent protein detectable in the NIR680 channel. Fluorescence intensity was quantified at the indicated time points to monitor treatment response. Data represent mean ± SD from three independent experiments. (G) Quantification of the fluorescence signal is shown as the change compared to day 1. *N* = 11, mean ± SD. One‐way ANOVA (Dunett's multiple comparison test), ***p < .001; ****p < .0001.

Functionally, THZ1 reduced proliferation (indicated by fewer pH3^+^ cells), and modestly increased PARP‐independent apoptosis (Figure S1C). Stemness markers remained largely unchanged, except for reduced SOX2 positivity in GBM15 (Figure S1C). Mesenchymal markers vimentin and N‐cadherin were unchanged, although a subset of E‐cadherin/N‐cadherin double‐positive cells emerged in GBM06 cells, suggesting partial phenotypic plasticity (Figure S1D).

We then assessed the impact of CDK7 inhibition on invasiveness using a 3D Matrigel assay (Figure 2D and E). THZ1 significantly reduced invasiveness of GBM spheroids in 2 of 3 cases over 10 days. Mevociclib, had similar effects (Figure S2C and D), confirming that CDK7 inhibition impairs GBM invasion in 3D culture. In a 3D‐bioprinted GBM model, THZ1‐treated scaffolds showed continuous viability loss over 14 days, unlike controls which stabilised after 6 days, demonstrating sustained cytotoxic effects (Figure 2F and G).

We subsequently evaluated THZ1 efficacy in PDOs generated from GBM tissue (*n* = 10, Table 1 and Figure 3). Only PDOs exhibiting > 80% viability by Calcein AM staining were included (Figure 3A and B, left). THZ1 reduced viability in most models; with statistically significant effects in approximately half (Figure 3C). Notably, GBM39, a rare subtype with primitive neuronal features,^9^ retained high viability despite morphological disintegration (Figure 3B and C), suggesting intrinsic resistance.

**FIGURE 3:**
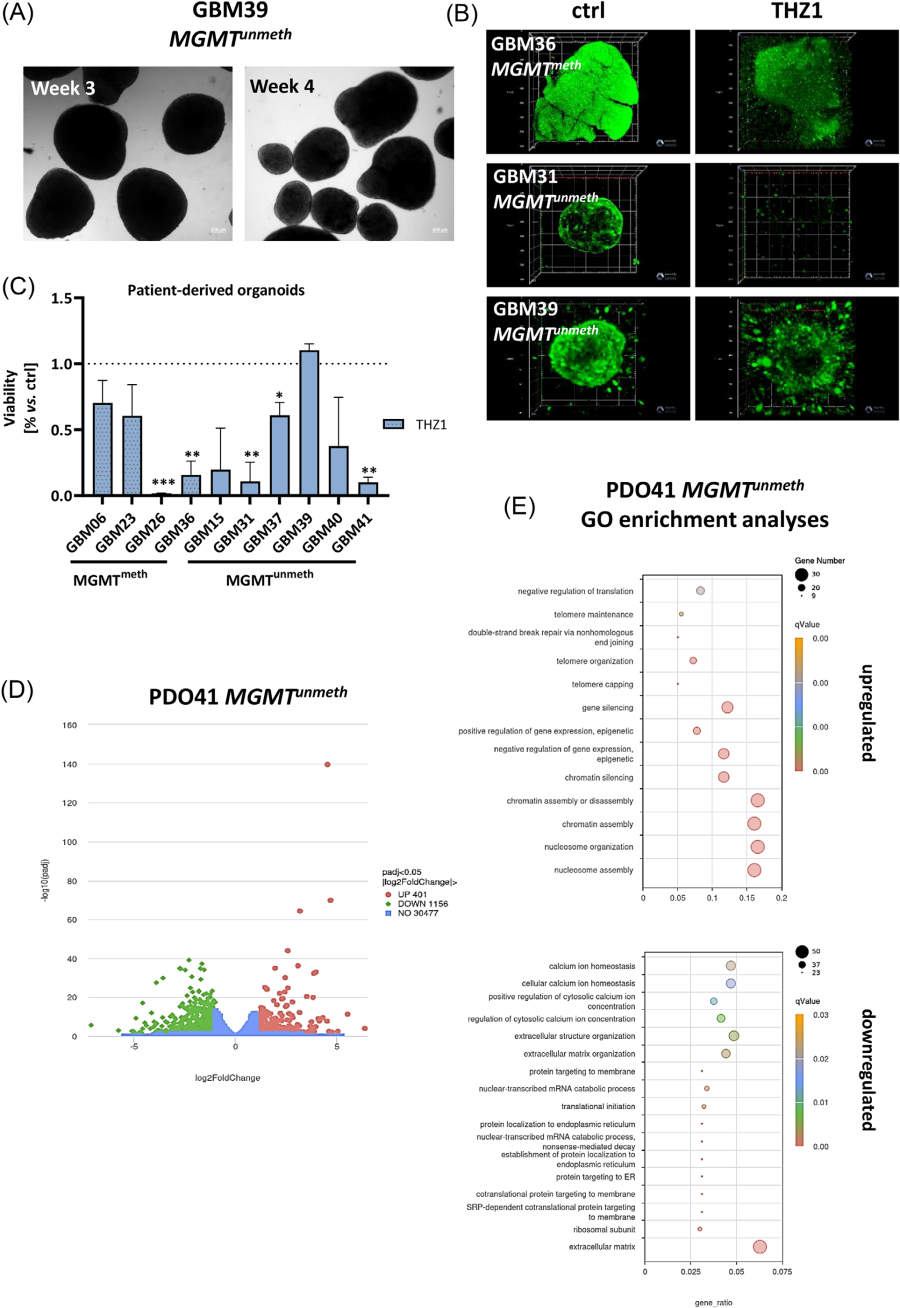
Effect of CDK7 blockade on viability and gene expression of patient‐derived organoids. PDOs were generated from fresh and frozen tissue samples of *n* = 10 individual GBM cases with or without MGMT promoter methylation. (A) Representative phase contrast images of PDOs at week 3 and 4 of culture in defined PDO medium. (B) Calcein AM staining was done to visualise the 3D‐structure of PDOs and to assess the viability via confocal laser scanning microscopy (Z‐stack analysis). Therefore, growing PDOs (approximately at day 35 of culture) were cut into defined pieces (∼200 µm), transferred into 96‐well ULA plates and exposed to THZ1 (100 nM). Confocal laser scanning microscopy was done after 10 days of treatment (including 2 × 72 h, + 4 days follow‐up). (C) Quantitative analysis of PDO viability was assessed after 10 days of treatment (including 2 × 72 h, + 4 days follow‐up) using the CellTiter‐Glo® 3D Cell Viability Assay as described in material and methods. *N* = 2–3 individual PDOs/case, mean + SD. *t*‐test; *p < .05; **p < .01; ***p < .001 vs. control. Dotted pattern – MGMT^meth^. (D) Volcano plot representing differentially expressed genes between control and the treatment group in PDO41 (THZ1 100 nM); log2 fold change > 1 or < 1 and a *p* adj < .05. (E) Significant upregulated and downregulated treatment‐associated regulation of GO terms; *q* < .05.

RNA sequencing of a sensitive case (PDO41) revealed 1557 differentially expressed genes (log_2_FC > 1 or < –1, adj. *p*  <  .05; Figure 3D). GO enrichment analysis showed upregulation of chromatin regulation and DNA repair, alongside downregulation of ribosomal function, calcium homeostasis, and mRNA decay (Figure 3E). Mitochondrial impairment was evident through reduced expression of genes involved in membrane integrity, coenzyme metabolism, and oxidative phosphorylation.

Transcriptomic analyses of PDO41 (THZ1‐sensitive) and PDO39 (THZ1‐resistant) identified distinct molecular pathways linked to sensitivity and resistance. In PDO41, THZ1 disrupted key biological processes, including protein synthesis, ribosomal function, cytosolic calcium ion concentration, and extracellular matrix organisation, indicating increased cellular vulnerability. GO analysis highlighted disruptions in nuclear‐transcribed mRNA catabolic processes, nonsense‐mediated mRNA decay, and responses to ER stress. DNA repair mechanisms, specifically double‐strand break repair via non‐homologous end joining and telomere maintenance, were upregulated, likely reflecting adaptive responses to genomic stress.

Conversely, PDO39 resistance was linked to upregulation of pro‐survival pathways, including cell cycle control, calcium signalling, and GABAergic synapses (Figure S3). Despite downregulation of canonical pathways like Wnt, TGF‐β, and MAPK,^10^ alternative routes – such as neuroactive ligand‐receptor interactions and TRP channel regulation – supported continued tumour growth. While adhesion and migration pathways were partly impaired, oxidative stress responses (e.g., UPR, ER stress) were evident, suggesting partial vulnerability and implicating mitochondrial dysfunction as a common mechanism upon CDK7 inhibition. Thus, prolonged CDK7 inhibition may represent a viable strategy to overcome resistance in such resilient clones.

In conclusion, CDK7 inhibition in GBM induces DNA damage, metabolic stress, and transcriptional disruption, leading to cell death. Resistance in some subtypes highlights the need for personalised, combination therapies targeting compensatory survival pathways.

## Supporting information



Supporting Information
