# Supplementary material for: CDK7 inhibition promotes glioblastoma cell death through convergent transcriptional and metabolic stress pathways
Source: Clin Transl Med. 2025 Aug 20;15(8):e70448. doi: 10.1002/ctm2.70448 (PMC12367859; doi:10.1002/ctm2.70448)
Supplement: Supplementary file 1 — Supporting Information [file CTM2-15-e70448-s001.pdf]

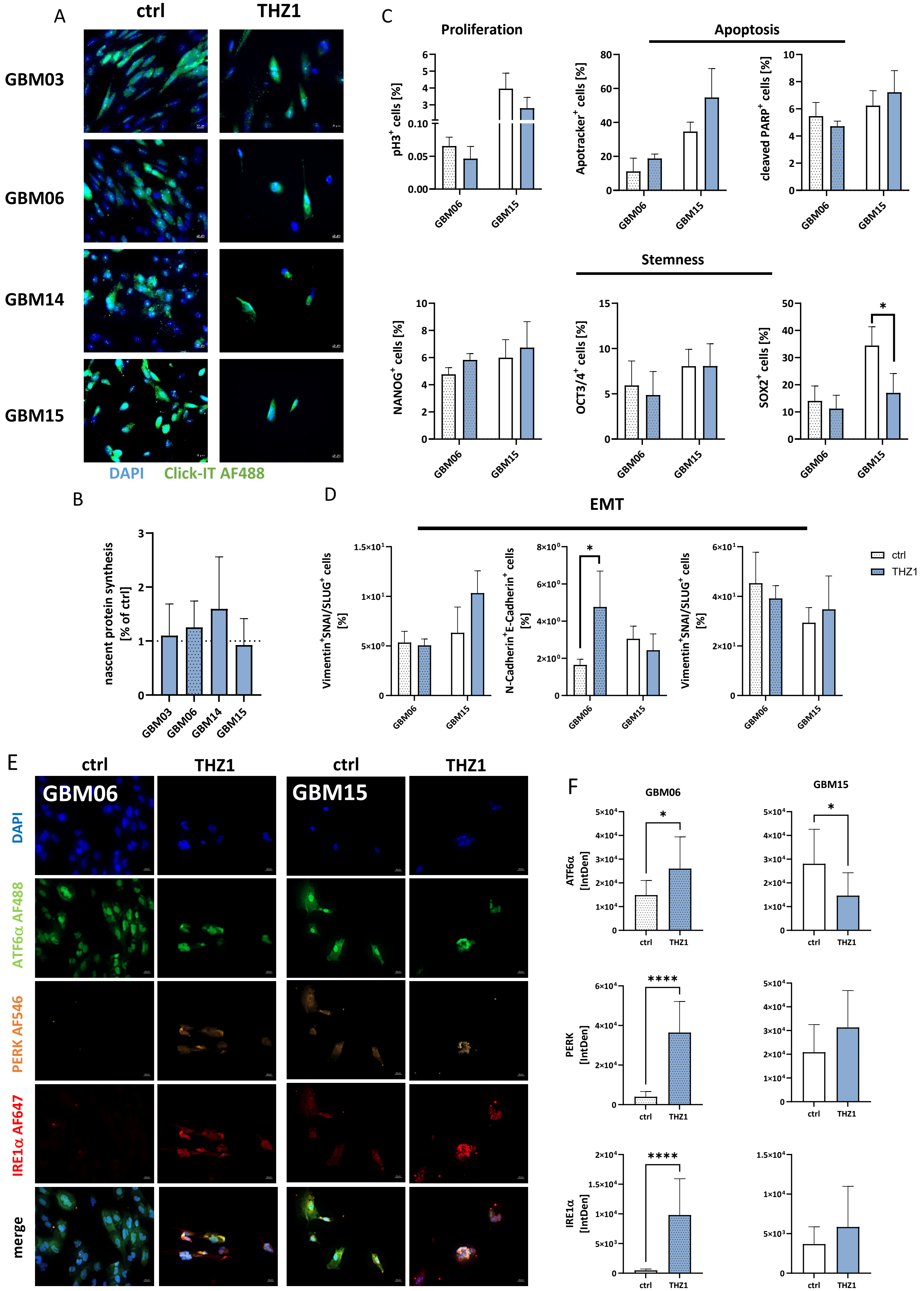

Supplementary Figure 1

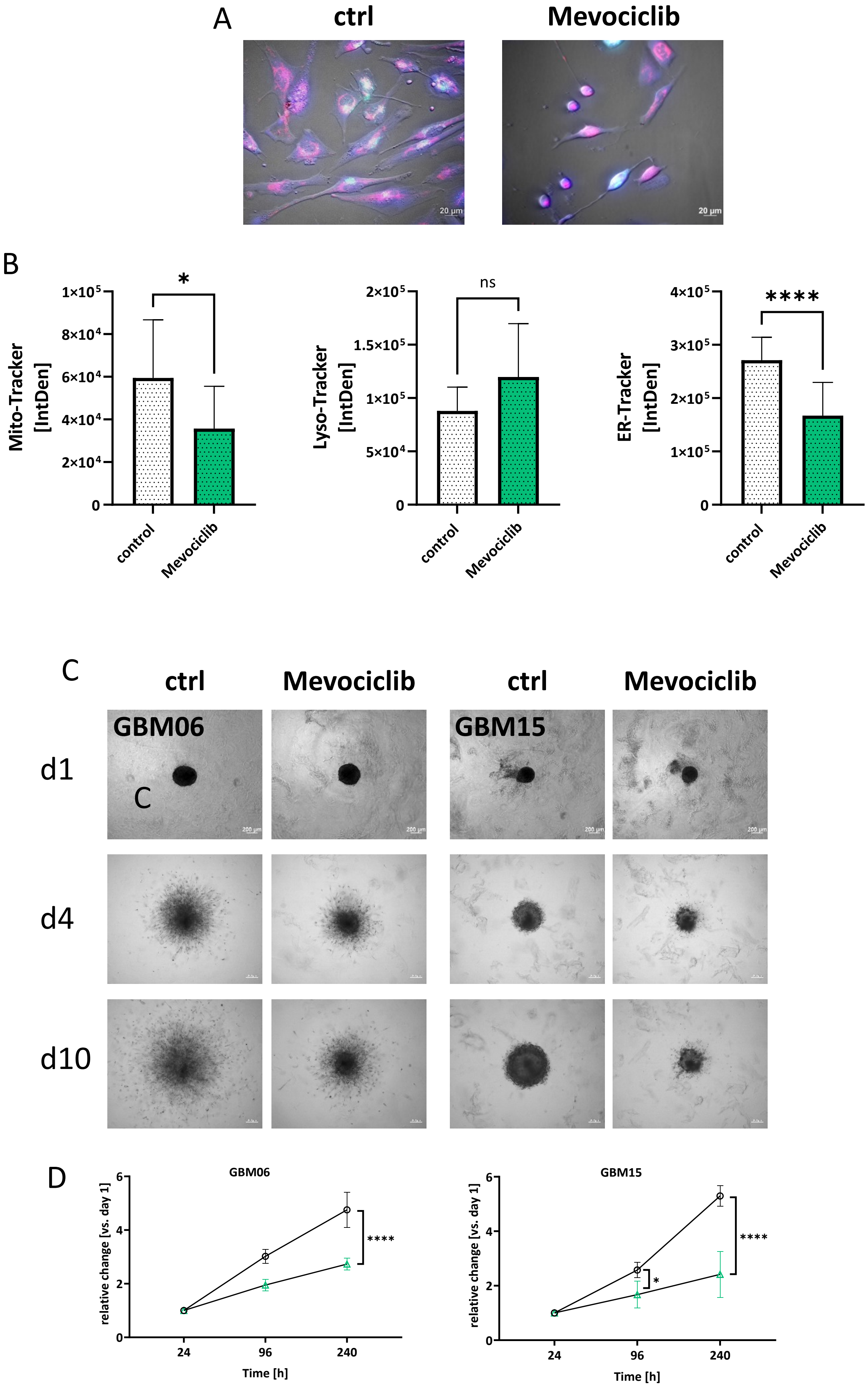

Supplementary Figure 2

A

PDO39 *vs.* PDO41

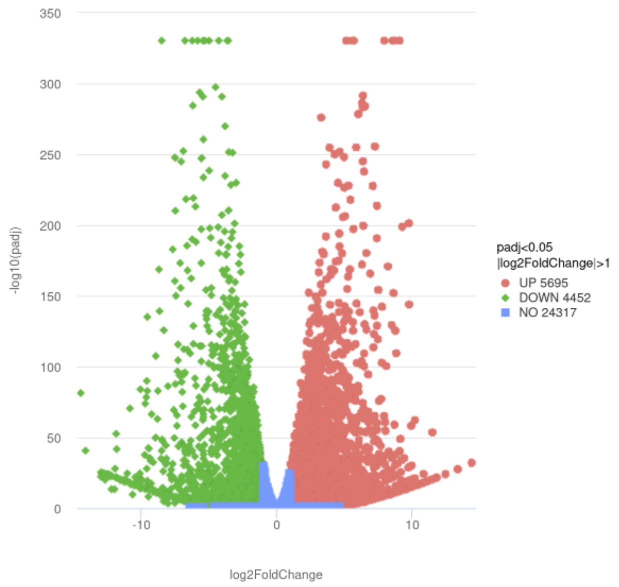

B

PDO39 *vs.* PDO41
KEGG enrichment analyses

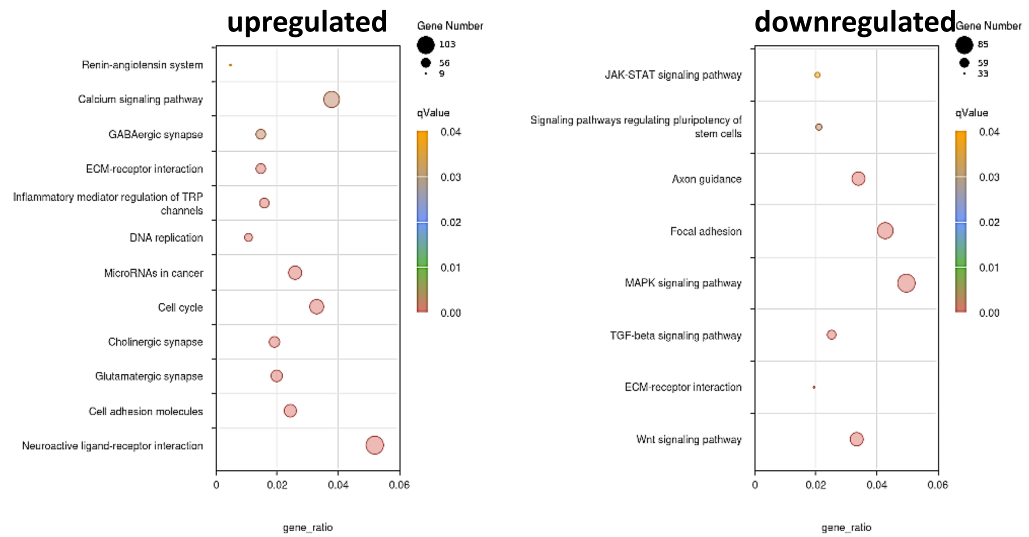

PDO39 *vs.* PDO41
GO enrichment analyses

C

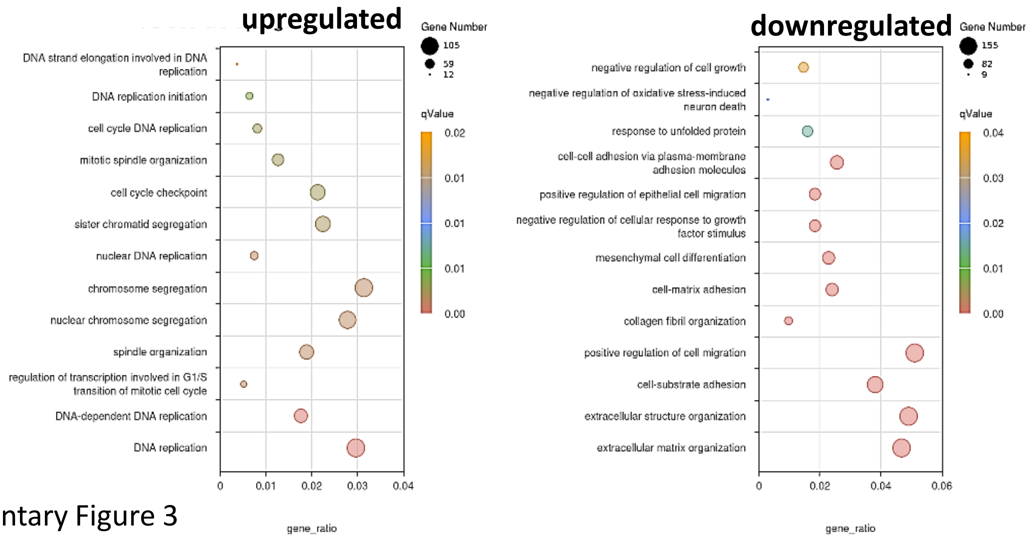

Supplementary Figure 3
